# Student Performance and Perceptions of Flipped Classrooms and Small Group Discussions as Teaching Tools in Practical Anatomy

**DOI:** 10.7759/cureus.68459

**Published:** 2024-09-02

**Authors:** Mohammad I Jumaa, Safaa M Hanafy, Karim H Farhat, Mostafa A Arafa, Mohamed Fawzi Farahat

**Affiliations:** 1 Department of Anatomy and Physiology, College of Medicine, Imam Mohammad Ibn Saud Islamic University, Riyadh, SAU; 2 Department of Surgery, The Cancer Research Chair, College of Medicine, King Saud University, Riyadh, SAU; 3 Department of Epidemiology, High Institute of Public Health - Alexandria University, Alexandria, EGY; 4 Department of Community Health Sciences, College of Applied Medical Sciences, King Saud University, Riyadh, SAU; 5 Department of Environmental Health, High Institute of Public Health - Alexandria University, Alexandria, EGY

**Keywords:** performance, students’ attitude, applied anatomy teaching, flipped classroom, medical education

## Abstract

Introduction

A quasi-experimental study was conducted to assess students’ attitudes toward the flipped practical classroom and evaluate its effectiveness in teaching practical anatomy.

Methods

Two survey questionnaires were developed: the first assessed students’ attitudes toward flipped practical classrooms, while the second focused on the obstacles and difficulties students might encounter in this learning environment.

Results

We found that students rated flipped learning significantly higher compared to other teaching methods, particularly in terms of the quality of learning materials, enhancement of learning skills, engagement and understanding of anatomy topics, and problem-solving abilities. The highest mean examination grades were observed for the pretest flipped modality, followed by the pretest small group discussion (mean scores: 82.72 vs. 54.46, F = 43.2, P = 0.004).

Conclusions

Students hold positive attitudes toward flipped classrooms and small group discussions compared to traditional classes.

## Introduction

Medical schools’ adoption of integrated curricula has led to a reduction in the size of gross anatomy lectures and cadaveric laboratories [1,2]. However, anatomists and clinicians assert that a precise and comprehensive understanding of human anatomy is essential for safe clinical practice [3]. Anatomy teachers are beginning to advocate for innovative teaching methods that enable students to actively engage with and apply critical clinical and factual knowledge within a shorter time [4-6].

Active learning provides students with more opportunities to solve problems and engage in critical thinking, which is more effective than the passive, teacher-centered approach of traditional lectures [7].

The flipped classroom is one of the cutting-edge pedagogical strategies that fosters student participation [8-10]. In this approach, the traditional roles of in-class and at-home tasks are reversed [11,12]. During the pre-class period, students are expected to self-direct their learning by reading textbooks, watching pre-made, brief instructional videos, and completing assignments or formative tests to assess their understanding [13-15]. Within the classroom, students are encouraged to engage in in-depth discussions through case-based learning problems, thereby advancing their learning objectives to higher cognitive levels [7].

Student-directed small group discussions are an additional active learning strategy that helps students enhance their knowledge, abilities, and professional and personal qualities [16,17]. These discussions include tasks that must be completed, such as identifying anatomical landmarks, structures, and functions. Resources such as eBooks, humans (classmates), artificial intelligence, notes, and search engines have been informally described as the peripheral brain [18].

The current study aimed to evaluate students’ attitudes toward flipped classrooms and assess the effectiveness of flipped classrooms, small group discussions, and traditional classrooms in teaching practical anatomy, as reflected in their final grades.

## Materials and methods

Inclusion and exclusion criteria

Second-year medical students were included in the study. Students from other years were excluded.

Study design

A quasi-experimental study design was employed.

Location

The study was conducted in the Department of Anatomy at Imam Mohammad Ibn Saud Islamic University, Riyadh, Saudi Arabia.

Study duration

The study took place from January to June 2023.

Study tools

The musculoskeletal system course integrated anatomy, embryology, physiology, and common diseases of the musculoskeletal system. It included 42 traditional lectures and 19 labs. The study focused on three core anatomy lab topics: triangles of the neck, scapular region and back, and vertebral column. Each topic was preceded by three traditional lectures. The topics were taught using three different methods: triangles of the neck through a flipped classroom, scapular region and back through small group discussions, and the spine using traditional methods. The flipped classroom and small group discussion modules were developed and approved by subject matter experts through peer review.

The second-year students were randomly divided into three groups: Group A (65 students), Group B (66 students), and Group C (67 students). Initially, Group A, Group B, and Group C were taught “triangles of the neck” using the flipped classroom approach. Subsequently, all three groups were instructed on the “vertebral column” using the traditional method. Finally, the “scapular region and back” were taught to the same groups through small group discussions, following the procedure specified in Table 1.

**Table 1:** Strategies of learning in anatomy lab sessions MCQ, multiple-choice question

	Flipped classroom	Traditional learning	Small group discussion
Before-class learning	Using cadaveric specimens, the researcher created short videos (less than 20 minutes) explaining the anterior and posterior neck triangles. Seven days before the planned class session, students were provided with access to the learning objectives, PowerPoint, and recorded video via Blackboard, the learning management system, so they could view it whenever they wished. Students can jot down any queries or ideas that were unclear while watching the video. After gathering and preparing the learning inquiries from students, the researcher selected those that were common and suitable for discussion.	Non-before-class activity	Non-before-class activity
Pretest formative assessment	An MCQ test was used to gauge students’ knowledge before in-class learning using Google Forms (10 MCQs in 10 minutes).	An MCQ test was used to gauge students’ knowledge before in-class learning using Google Forms (10 MCQs in 10 minutes).	An MCQ test was used to gauge students’ knowledge before in-class learning using Google Forms (10 MCQs in 10 minutes).
In-class learning	A quick mini-session was held to go over the key points of the pre-class readings. Answering questions from students: asking questions to encourage teacher-student and student-student interaction.	The topic and learning goals were presented using a standard traditional method.	Each group was subdivided into small subgroups (about 11 students each). At the beginning of the session, the objectives were explained to the students, and they were provided with the learning resources (atlases of human anatomy, a PowerPoint presentation, and cadaveric specimens of human back and shoulders). A leader and scribe were identified within each subgroup. Each subgroup was tasked to accomplish a checklist of structures, landmarks, and functions and was then asked to discuss the required outcomes as a group with instructor supervision.
Post-class	Post-test formative assessment: An MCQ test was used to gauge students’ knowledge before in-class learning using Google Forms (10 MCQ in 10 minutes). A survey was used to get student feedback.	Post-test formative assessment: An MCQ test was used to gauge students’ knowledge before in-class learning using Google Forms (10 MCQ in 10 minutes). A survey was used to get student feedback.	Post-test formative assessment: An MCQ test was used to gauge students’ knowledge before in-class learning using Google Forms (10 MCQ in 10 minutes). A survey was used to get student feedback.

Two survey questionnaires were developed to evaluate students’ attitudes toward flipped classrooms during anatomy laboratory sessions using a structured, pre-designed tool.

The first survey addressed four domains: the role of flipped classrooms in general learning and specifically in practical anatomy, pre-class activities, problem-solving, and the Objective Structured Practical Examination (OSPE) examination. Additionally, it included a question soliciting students’ opinions on improving the flipped classroom for practical teaching (Appendix A).

The second survey focused on identifying obstacles and difficulties students might face in practical flipped classrooms and their perceptions of the three different teaching methods. The attitude scale comprised 10 questions on a 5-point Likert scale, ranging from 5 (strongly agree) to 1 (strongly disagree). Students’ final grades across different teaching modalities were recorded and compared. The survey was piloted with 15 students, resulting in a Cronbach’s alpha of 0.91. Preferences and perceptions were assessed using nine questions regarding aspects such as the quality of learning materials, enhancement of learning skills, engagement with anatomy topics, problem-solving ability, and the impact of examination and discussion throughout the course (Appendix B). Surveys were conducted via Google Forms, with students accessing the link through WhatsApp.

Ethical approval for the study was obtained from the Ethics Committee of the Faculty of Medicine, Imam Mohammed bin Saud Islamic University, Riyadh, Saudi Arabia (approval number 367/2022).

Statistical tests

Data were analyzed using IBM SPSS Statistics for Windows, Version 26.0 (Released 2019; IBM Corp., Armonk, NY, USA). The percentage responses were calculated based on the mean and range of total responses for each item. Categorical variables were compared between groups using the chi-square test. For continuous variables, the Student’s t-test and F-test were employed. The Z test was used to compare proportions for different items on the attitude scales. A P-value of less than 0.05 was considered statistically significant.

## Results

A total of 198 students participated in the study, with 138 (69.7%) being male. The students showed a positive attitude toward the flipped practical classroom, with a mean total attitude score of 39.5 ± 6.4 (79%). Responses of “strongly agree” and “agree” were combined, as were those of “strongly disagree” and “disagree.” No significant difference was found between males and females: the mean total attitude score for males was 39.2 ± 3.1, while for females it was 40 ± 8.7 (t = 0.76, P = 0.4).

In this study, 149 students (75.25%) reported that they were exposed to a flipped classroom for the first time.

Table 2 presents the distribution of students’ attitudes toward flipped practical classrooms across four domains. The students expressed a highly positive attitude in all domains. They acknowledged the significant role of flipped education, particularly the effectiveness of well-trained teaching staff, the positive impact on the OSPE exam, and improvements in problem-solving skills and clinical contexts. The positive attitude (combining “strongly agree” and “agree”) exceeded the negative attitude (combining “strongly disagree” and “disagree”) by a range of 67.2-80.3%, highlighting the perceived value of the flipped classroom approach.

**Table 2:** Distribution of attitude statements toward flipped classrooms among students * a significant p-value ≤ 0.05 OSPE, Objective Structured Practical Examination

Domains	Attitude statements	Strongly agree and agree, N (%)	Strongly disagree and disagree, N (%)	Z score	P
Role of flipped classes for learning in general and for practical anatomy in specific	The flipped classroom approach was valuable and interesting for engagement in practical anatomy sessions.	149 (75.25)	11(5.5)	14.04	0.0001*
	The flipped classroom is a simple and comfortable tool for learning practical anatomy.	150 (75.8)	13 (6.6)	3.1	0.003*
	Was your ability to use new learning technologies improved by the flipped classroom?	138 (69.7)	11 (5.6)	13.1	0.002*
	Do you encourage flipped classrooms as a learning tool in the anatomy lab?	154 (77.8)	14 (7.1)	14.2	0.003*
Pre-class activity	Is it beneficial to watch a demonstration video before the class?	153 (77.27)	17(8.5)	14.9	0.0003*
	The pre-class activity is a helpful tool to support the understanding of practical topics.	162 (81.8)	9 (4.5)	15.4	0.002*
	The pre-session preparation facilitates good discussion during class time.	146 (75.7)	8 (4)	15.2	0.001*
Problem-solving and OSPE examination	The problem-solving ability and clinical correlation have been enhanced with the flipped classroom.	142 (71.7)	9 (4.5)	13.5	0.001*
	The flipped classroom is helping to prepare students for the OSPE examination in comparison with the traditional practical session.	156 (78.8)	9 (4.5)	15	0.001*
Improvement of flipped classes	Do you think that qualified teaching staff and well-prepared teaching material can improve the flipped classroom?	168 (84.8)	9 (4.5)	14.5	0.002*

Table 3 shows the mean differences between pre-test and post-test examination grades for various teaching modalities. No significant difference was found for the practical flipped education (t = 0.58, P = 0.45). However, significant differences were observed for practical traditional learning and practical small group discussions, with t-values of 9.7 (P = 0.0001) and 3.3 (P = 0.01), respectively. The greatest difference was noted between practical traditional learning and practical small group discussions, with mean differences of 34.1 points and 15.5 points, respectively.

**Table 3:** Paired t-test for pre-test and post-test examination results for different teaching modalities * a significant p-value ≤ 0.05

Teaching modality	Test	Mean (SD)	T-test	P
Practical flipped	Pre-test	80.74 (25.3)	0.58	0.45*
	Post-test	82.72 (19.9)		
Practical small group discussion	Post-test	52.1 (31.1)	3.3	0.01*
	Post-test	67.6 (25.3)		
Practical traditional	Post-test	50.5 (24.1)	9.7	0.0001*
	Post-test	84.7(19.9)		

Table 4 displays the differences in mean grades between pre-test and post-test examinations across the three educational modalities. Significant differences were observed among the modalities. For the pre-test examination, the highest mean was recorded for flipped education, followed by small group discussion (mean ± SD = 82.72 ± 19.9 and 54.48 ± 24, respectively; F = 43.2, P = 0.004). For the post-test examination, traditional education yielded the highest mean, followed by flipped education (mean ± SD = 84.4 ± 19.6 and 80.2 ± 26.2, respectively; F = 8.9, P = 0.002).

**Table 4:** Means of the examination grades for the three modalities of education * a significant p-value ≤ 0.05

	Examination modality	Mean	Standard deviation	F	P
Pre-test	Small group discussion	54.46	31.326	43.2	0.004
	Flipped	82.72	19.938		
	Traditional	49.71	24.069		
Post-test	Small group discussion	67.59	25.361	8.9	0.002
	Flipped	80.22	26.269		
	Traditional	84.47	19.61		

Table 5 presents the comparison of mean differences in examination grades across the three educational modalities. For pre-test examination grades, the most significant mean difference was observed between flipped and traditional education (33, P = 0.0002), followed by flipped versus small group discussion (28.2, P = 0.001). For post-test examination grades, the greatest and most significant mean difference was between traditional and small group discussions (16.8, P = 0.002), followed by flipped versus small group discussion (12.6, P = 0.05).

**Table 5:** Mean difference comparisons of the examination grades for the three modalities of education * a significant p-value ≤ 0.05

	Examination modality comparisons	Mean difference	P
Pre-test	Flipped vs. traditional education	33	0.0002*
-	Flipped vs. small group discussion	28.2	0.001*
Post-test	Traditional vs. small group discussion	16.8	0.002*
-	Flipped vs. small group discussion	12.6	0.05*

**Figure 1:**
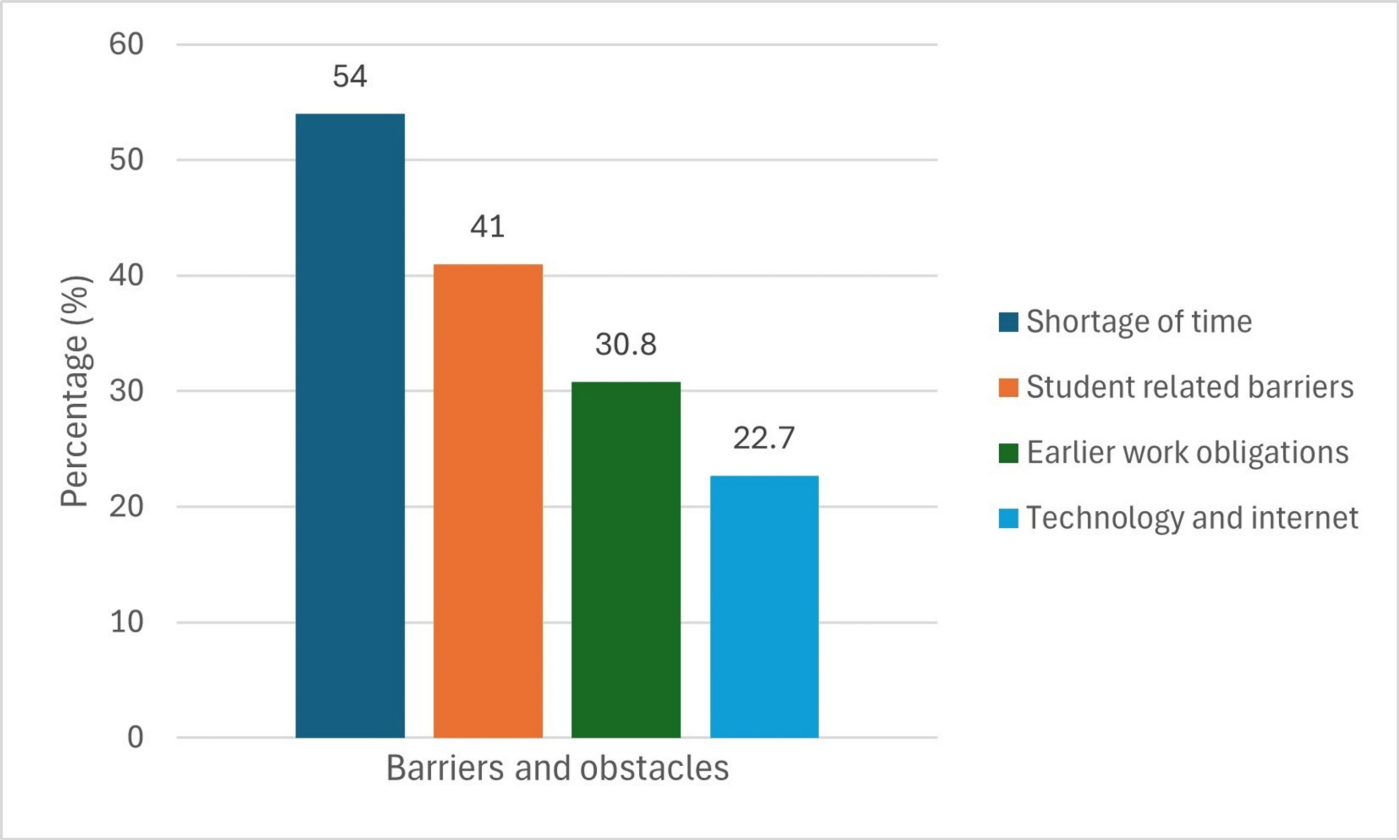
Barriers and obstacles expressed by students toward the practical flipped classroom

Figure 1 illustrates the primary obstacles and barriers to practical flipped classrooms reported by students. The most common issue was a shortage of time, affecting 107 students (54%). This was followed by student-related barriers (81 students, 41%), prior work obligations (61 students, 30.8%), and technology and internet issues (45 students, 22.7%).

Students perceived flipped learning as superior to other teaching techniques, with small group discussions also rated higher than traditional learning. This preference was particularly evident regarding the quality of learning materials, enhancement of students’ learning skills, engagement, understanding of anatomy topics, and problem-solving ability. The most significant differences in opinion were noted in the areas of exam preparation and course discussions, with 42% and 23.3% favoring flipped learning, respectively (Table 6).

**Table 6:** Comparison between the three techniques of teaching based on students’ preferences and perceptions

Students’ opinion	Small group discussion, N (%)	Flipped learning, N (%)	Traditional learning, N (%)
Which was increasing the student’s interest and level of engagement in the anatomy lab?	59 (29.8%)	120 (60.6%)	19 (9.6%)
Which was more helpful in understanding the anatomy topics?	52 (26.3%)	125 (63.1%)	21 (10.6%)
Which improves the quality of learning materials?	49 (24.7%)	132 (66.7%)	17 (8.6%)
Which improves the student's learning skills?	87 (43.9%)	96 (48.5%)	15 (7.6%)
Which increases student support and competencies?	65 (32.8%)	112 (56.6%)	21 (10.6%)
Which enables the student to have a good discussion during the course?	66 (33.3%)	112 (56.6%)	20 (10.1%)
Which improves the student’s problem-solving ability and clinical correlation?	80 (40.4%)	93 (47%)	52 (12.6%)
Which was better for preparing the students for the examination?	46 (23.2%)	129 (65.2%)	23 (11.6%)

## Discussion

The inverted classroom, also known as the flipped classroom, reverses the traditional learning model: lectures are assigned as homework, while activities previously done at home are now completed in class [19]. In this study, 75.25% of students reported they had never experienced a flipped classroom. This finding is consistent with research indicating that 80% of the reviewed literature on flipped classrooms focuses on higher education [11].

Furthermore, 75.8% of students in this study found the flipped classroom method comfortable for learning practical anatomy, and 75.25% believed it enhanced their class participation. These results are in line with El Sadik and Al Abdulmonem, who found that 40% of students found the inverted classroom model engaging [20]. Other studies have shown that flipped classroom tools increased student satisfaction by 18% [21] and engagement by 14% [22].

Our study found that 69.7% of students believed flipped classes improved their ability to utilize new learning tools, which aligns with El-Ashkar et al.’s observation that advanced technologies in flipped classrooms are highly valued for innovative learning [23]. Most students (77.7% and 81.8%) felt that pre-class demonstration videos were beneficial for enhancing their understanding of practical anatomy topics. This is consistent with El-Ashkar et al., who found that pre-class assignments significantly enhanced students' understanding of practical parasitology [23]. Similarly, El Sadik and Al Abdulmonem reported that flipped classrooms improved medical students’ ability to understand and analyze anatomy materials [20].

Students showed the most favorable attitudes toward the flipped classroom in relation to OSPE examinations, problem-solving ability, and clinical correlation. This supports El Sadik and Al Abdulmonem’s finding that flipped learning and peer tutoring encourage deeper engagement in critical thinking and problem-solving [20].

However, the study identified several challenges to implementing flipped classrooms. The primary obstacle was a lack of time, reported by 54% of students, followed by student-related barriers (41%), including difficulties with self-regulation. Early work commitments (30.8%) and technology and internet barriers (22.7%) were also significant issues, highlighting disparities in technology access. Previous studies have noted that flipped classrooms can be time-consuming and challenging to manage, with both educators and students needing advanced technological skills [24-27].

One of the key findings was the comparison of student grades across different teaching modalities. For pre-test examinations, the highest mean score was observed in the flipped classroom, followed by small group discussions, aligning with Van Wyk’s findings that pre-test grades in flipped classrooms were higher than those in traditional settings [28]. Conversely, for post-test examinations, traditional education yielded the highest mean score, followed by flipped education. This discrepancy might be due to variations in exam difficulty. Tsao et al. found that while both traditional and flipped classroom methods enhance academic achievement, the flipped approach generally surpasses traditional lectures [29].

Overall, students rated flipped learning significantly higher than other methods, with small group discussions also preferred over traditional learning. This preference was especially evident in aspects such as the quality of learning materials, skill enhancement, engagement, and problem-solving. These findings are supported by Bhavsar et al., who observed that flipped classrooms enhance learning, critical thinking, and examination performance in medical education [30].

Limitations

This study has some limitations. First, a significant number of students did not adhere to the instructions or follow their mentors’ guidance for the flipped classroom approach. Second, the absence of a focus group discussion before the study limited our ability to capture student challenges and gather detailed suggestions for improvement.

## Conclusions

The flipped classroom and small group discussions prove to be more effective methods for learning practical anatomy compared to traditional classes, as evidenced by students’ positive attitudes and improved examination grades. Enhancing and encouraging the use of these methods in practical anatomy sessions and other basic courses in medical colleges is recommended. However, despite its effectiveness, implementing the flipped classroom approach presents challenges, especially in regions where students are accustomed to traditional teaching methods and may be reluctant to commit additional time to flipped learning. Addressing these challenges will require collaborative efforts from students, faculty, and administration within educational institutions.

## Appendices

 Appendix A

**Table 7:** Survey to Assess Student Attitudes to the Flipped Classroom in Practical Anatomy

	Items	Strongly agree	Agree	Neutral	Disagree	Strongly disagree
1	Do you think the flipped classroom approach was valuable and interesting for engagement in practical anatomy sessions?					
2	Do you think the flipped classroom is a simple and comfortable tool for learning practical anatomy?					
3	Was your ability to use new learning technologies improved by the flipped classroom?					
4	Do you encourage flipped classroom as a learning tool in anatomy lab?					
5	Is it beneficial to watch a demonstration video prior to the class?					
6	Do you think the pre-class activity is a helpful tool to support the understanding of practical topics?					
7	Do you think the pre-session preparation facilitates good discussion at the class time?					
8	Do you think the Problem-solving ability and clinical correlation have been enhanced with the flipped classroom?					
9	Do you think the flipped classroom is helping to prepare students for OSPE examination, in comparison with the traditional practical session?					
10	Do you think that qualified teaching staff and well-prepared teaching material can improve the flipped classroom?					

 Appendix B

**Table 8:** Survey to compare small group discussions, flipped learning, and traditional learning

	Items	Small group discussions	Flipped Learning	Traditional Learning
1	Which increased the students' interest and level of engagement in the anatomy Lab?			
2	Which was more helpful in understanding the anatomy topics?			
3	Which improves the quality of learning materials?			
4	Which improves the student learning skills?			
5	Which increases the student support and competencies?			
6	Which enables the student for good discussion during the course?			
7	Which improves the student Problem-solving ability and clinical correlation?			
8	Which was better for preparing the students for examination?			
